# Ethnic diversity outpatient clinic in paediatrics

**DOI:** 10.1186/1472-6963-12-12

**Published:** 2012-01-11

**Authors:** Nordin Dahhan, Dominique Meijssen, Malika Chegary, Diederik Bosman, Bart Wolf

**Affiliations:** 1 Dept of Paediatrics, St Lucas Andreas Hospital, PO Box 9243, 1006 AE Amsterdam Netherlands; 2 Dept of Paediatrics, Emma Children Hospital/Academic Medical Center, PO Box 22660, 1100 DD Amsterdam, Netherlands; 3 Dept of Paediatric Rehabilitation, Academic Medical Center, PO Box 22660, 1100 DD Amsterdam, Netherlands; 4 Dept of Paediatrics, Onze Lieve Vrouwe Gasthuis, PO Box 95500, 1090 HM Amsterdam, Netherlands

## Abstract

**Background:**

The health status of chronic sick ethnic minority children in the Netherlands is unequal compared with indigenous Dutch children. In order to optimize the health care for these children a specific patient-oriented clinic in ethnic-cultural diversity: the Mosaic Outpatient Clinic (MOC) was integrated in the general Paediatric Outpatient Departments (POPD) of three hospitals in Amsterdam.

**Methods:**

Feasibility of the MOC, factors influencing the health care process and encountered bottlenecks in health care were studied in ethnic minority children with asthma, diabetes type 1 or metabolic disease originating from Morocco, Turkey and Surinam. Feasibility was determined by the number of patients attended, support from the paediatric medical staff and willingness of the patients to participate. Influences on the health care process comprised parents' level of knowledge of disease, sense of disease severity, level of effort, linguistic skills, health literacy, adherence to treatment and encountered bottlenecks in the health care process. Moreover, the number of admissions and visits to the POPD in the years before, during and after the MOC were analysed.

**Results:**

In 2006 a total of 189 ethnic minority children were seen. Integration of the MOC within the general POPD of the hospital is feasible. The ability of the parents to speak and understand Dutch was found to be 58%, functional health literacy was 88%; sufficient knowledge of disease and sense of disease severity were 59% and 67%, respectively.

The main bottlenecks in the healthcare process: poor knowledge of disease, limited sense of disease severity and low health literacy in the parents proved to be the best predictors for decreased adherence. After attending the MOC there was a decrease in the number of admissions and visits to the POPD for asthma while the number of visits increased in patients with diabetes and the amount of no-shows decreased in patients with a metabolic disease.

**Conclusion:**

Integration of a MOC in the general POPD is feasible and appreciated by the parents, provides more insight in the problems ethnic minority children and their parents face and shows promising directions for optimizing adherence in these children.

## Background

In Amsterdam half of the population and nearly 2/3 of the children have a non-indigenous Dutch background; the largest groups are originating from Morocco, Turkey and Surinam [1]. These numbers are based on country of birth and comprise first generation migrants and their children [2]. The health status between ethnic minority and native Dutch children is unequal and both the perinatal and child mortality in the Netherlands is higher in ethnic minority children compared with indigenous Dutch children; at the same time a higher incidence of specific chronic hereditary diseases (i.e. hemoglobinopathies) is encountered in ethnic minority children [3]. Moreover, a recent Dutch study showed that ethnic minority children receive less frequent medical care and that diagnoses are made relatively late compared to indigenous Dutch children [4]. These differences may be explained by a poor information exchange between doctors and patients and their parents, due to language difficulties and/or a different cultural background [5,6]. The low socioeconomic status of many of these families however, may also negatively affect their health status [7]. Health disparities can be alleviated, in part, by creating and maintaining culturally competent healthcare systems [8].

In order to promote better and more satisfactory health care services to chronic sick ethnic minority children a profound analysis of the main constraints in the health care process is necessary. With this in mind, a specific MOC was introduced in the general Paediatric Outpatient Department (POPD) of three hospitals in Amsterdam. Aim of the MOC was to provide insight in the specific health care problems of chronic sick ethnic minority children and their parents in order to optimize the care for these children, and to identify bottlenecks in the health care process. Moreover, the feasibility of the MOC and factors influencing the health care process, in particular adherence, were studied. Finally the effect of the MOC on the use of hospital facilities was analysed retrospectively in a small pilot. The introduction of the MOC was evaluated in three diagnostic categories of children i.e. asthma, diabetes mellitus type 1 and metabolic disease, as these are demanding conditions that necessitate children and their parents to take extensive responsibility in its management. Asthma is the most frequent chronic disease in childhood and poorly controlled patients are prone for exacerbations and therefore increased admissions and unscheduled visits to the POPD and Emergency Department (ED) [9]. Diabetes type 1 is a chronic disease with a high incidence in children from Moroccan descent and regular visits to the POPD are important as frequent check-ups reduce long term complications [10,11]. Metabolic diseases, with its heterogeneous origins, are complex disorders that need regular follow-up as well.

We assumed that the establishment of the MOC in the general POPD would identify relevant bottlenecks in the health care process of chronic sick ethnic minority children and show directions for optimizing health care.

## Methods

### Patients

Ethnic minority children with chronic diseases like asthma, diabetes mellitus type 1, metabolic disease (inborn errors of metabolism), chronic abdominal pain, nephrological disorders, chronic headache, severe obesity or epilepsy, who were treated in two District Teaching hospitals (the St Lucas Andreas Hospital and the Onze Lieve Vrouwe Gasthuis) or in one University hospital (the Emma Children's hospital/Academic Medical Centre) in Amsterdam, the Netherlands, were referred to the MOC in 2006, if they had visited the POPD of their hospital in the past year more frequently than children with the same chronic disorder and/or when the health care process was felt non-optimal by their own paediatrician; patients with diabetes mellitus type 1 or a metabolic disease were all referred. The concerned patients and their parents were invited to participate through an informing letter sent by mail. The patient's parents were subsequently phoned by a health care worker from the MOC. Only after parents confirmed that they fully understood the aim of the MOC, informed consent to participate was requested. Although the respective Medical Ethical Committees from the participating hospitals were informed, formal approval was not sought as the Mosaic program was not a research but a health services evaluation project. As a sample data from the patients who were seen in the MOC during the initial year (2006) were analysed in this paper.

### The Mosaic Outpatient Clinic

The Mosaic Outpatient Clinic (MOC) is an integrated and patient-centered 2^nd ^line outpatient clinic in ethnic-cultural diversity within the POPD of the hospital and is easily accessible to the patients and their parents. Support is offered by a trained team of health care professionals existing of 2 supervising consultant paediatricians, 4 student healthcare workers as cultural mediators and a POD assistant. Mediators serve as the interface, translating language and interpreting culture, between the health care provider and the ethnic minority patient. In the UK 'health advocates' translate during consults but also educate clients and clinicians and in Belgium intercultural mediators try to solve problems minorities encounter while seeking health [12,13]

We integrated student healthcare workers, medical or psychology students with several ethnic backgrounds, who were trained in workshops by the two supervising paediatricians to perform independently a patient-centred consultation and to explore cultural sensitive issues and bottlenecks in the health care process. In preparation of the initial consultation at the MOC a complete medical history, use of medication, number of admissions and the number of visits to the POD and ED in the previous year (2005) were taken from the medical records. Moreover, data on demography, family situation and concepts and experiences about health and disease of parents were registered. At the first consultation at the MOC parents were asked in an open and participatory way to specify which health care problems they experienced with their children and how the care could be optimized. Moreover a profile of the parents was made by assessing their level of knowledge of the disease, sense of disease severity, adherence to treatment, level of effort, Dutch language skills and functional health literacy. The initial consultation took approximately 45 minutes. In general patients were seen 3-4 times in the MOC before they were referred back with a written report to their own paediatrician at the general POPD.

After a systematic inventory of the possible problems in the health care process, an individually tailored treatment plan was proposed by the healthcare worker varying from follow-up consultations at the MOC to increase knowledge of disease and self-management strategies, to referral to a child psychologist, dietician, paediatric physical therapist, social worker and/or any care delivery system. The proposed plan was fine-tuned with the supervising paediatrician and subsequently discussed with the patients and their parents by the healthcare worker and supervisor together.

### Measurements

The feasibility of the MOC was determined by four conditions: i) the number of patients attended, with a minimum of 200 per year in the three hospitals together, ii) support from the medical staff, iii) willingness from patients to participate and iv) financial sustainability.

Influences on the health care process were studied by the parents' profile and the identified bottlenecks by the Mosaic team. The profile of the parents was determined by their level of knowledge of disease, sense of disease severity, adherence to treatment, level of effort, Dutch language skills and their functional health literacy (Table 1), which were measured by the health care worker on a 4-point Likert scale: very low/bad, low/bad, medium or high/good. Moreover, for the level of effort an 'adequate' or 'inadequate' qualification was added, to indicate whether the level of effort of the parents was consistent with the severity of the medical condition. An adequately low level of effort represents a normal, suitable level of effort in relation to a low severity of the disease. An adequately high level of effort is seen in parents who give all the necessary care in relation to a high severity of the disease. An inadequately high level of effort is encountered in parents who are over-concerned and parents with an inadequately low level of effort are considered not to be able to manage their child's disease in a responsible way. The first 30 patients and their parents were assessed by the entire Mosaic healthcare team to come to equal scores for the above-mentioned profiles. Interrater reliability was more than 90%. Moreover, all patients were seen by only 2 supervising paediatricians (ND and BW) increasing the reliability of the scores. Finally, to gain insight of the MOC on the use of hospital facilities, the number of admissions and visits to the POPD and ED in the years before (2005), during (2006) and after (2007) the MOC was introduced, were analysed retrospectively.

**Table 1:** Definitions of parents' profiles

Knowledge of disease	The knowledge about the characteristics of the disease
Sense of disease severity	The knowledge about possible complications of the disease
Adherence	Correct use of prescribed medicine and adherence to treatment advice (e.g. diet/inhalation therapy)
Level of effort	The level of effort in relation to the severity of the disease
Dutch language skills	The ability to speak and understand Dutch (good is if communication is possible without any linguistic barriers, e.g. an interpreter-translator)
Functional health literacy	The ability to understand and correctly use information during the consultation (e.g. giving a rationale of the prescribed treatment)

### Statistical analyses

Descriptive statistics were applied to describe the 3 main ethnic minority groups (i.e. Moroccan, Turkish and Surinamese). Analyses were done for the characteristics of the total group of parents, except for Dutch language skills in Surinam parents, as people from Suriname speak and understand Dutch fluently. Spearman's rho was used for assessing correlations between language skills, health literacy, knowledge of disease, disease severity, effort level and compliance in the total group and in subgroups to compare the Moroccan and Turkish parents. Logistic regression was used to determine the best predictor variables for compliance (very low to moderate compliance = 0, good compliance = 1).

Analyses of variance for repeated measures were used to compare the use of hospital facilities in the three main diagnostic categories (asthma, diabetes mellitus type I and metabolic disease). In the two district hospitals (St Lucas Andreas Hospital and Onze Lieve Vrouwe Gasthuis) the number of admissions, visits to the POPD and ED in the asthma and diabetes category before, during and after the Mosaic intervention were compared. As children with metabolic disorders in Amsterdam are all treated in the University Hospital (Emma Children's hospital/Academic Medical Centre), the use of that hospital facility was measured by the number of admissions, visits to the POPD, consultations by telephone and "no shows" at the POPD.

## Results

Since the start of the MOC, approximately 750 new patients were seen in the past five years (2006-2010). In this study only patients from the initial year (2006) are included when a total of 189 patients and their parents were seen in the MOC's of the three participating hospitals. Patient's ethnic backgrounds were Moroccan (n = 100; 53%), Turkish (n = 51; 27%), Surinam (n = 13; 7%) or "other ethnic minority" (n = 25; 13%). There were 119 patients in the 3 main diagnostic categories: 58 patients with asthma (mean age 5.3 years, SD 4.6 years), 28 patients with diabetes type 1(mean age 11.2 years, SD 3.8 years) and 33 patients with a metabolic disease (mean age 8.2 years, SD 5.1 years).

### Feasibility of the MOC

The estimated amount of patients to be seen in the MOC's of the three hospitals was achieved; over 95% of the parents were willing to participate and more than 90% of the parents proved to be satisfied with the MOC. The initial inclusion of the patients was hampered due to unfamiliarity with and confusion about the MOC; some patients regarded the MOC as a permanent alternative to their regular paediatric outpatient care and some paediatric staff felt sceptic about the initiative. However, the outline of the MOC became clearer after logistic adaptations (e.g. separate appointment system) and intensifying of the information about the MOC to patients, parents and staff. Moreover the MOC proved to be financially sustainable and could be easily implemented and sustained without extra funding after an initial incentive from the local Health Insurance Company to start the project. Although we did not perform a cost-effective analysis the management of the hospitals agreed in continuing and integrating the MOC in the regular hospital services after analysing the MOC results.

### Influences on the health care process

#### Parents' profiles

The ability to speak and understand Dutch in the group of 189 migrant parents was sufficient in 58% and health literacy proved to be sufficient in 88% (Table 2). Moroccan parents showed better Dutch language skills than Turkish parents (X^2 ^12.8, p < 0.01). However, no differences were found in health literacy between both groups (X^2 ^4.0, p = 0.26).

**Table 2:** Parents' profile in the Mosaic Outpatient Clinic

N = 189	Very bad/low	Bad/low	Moderate	Good/high
Knowledge of disease	11.3%	29.9%	32.8%	26%
Sense of disease severity	9.3%	21.5%	13%	56.2%
Adherence	1.1%	11.9%	14.7%	72.3%
Dutch language skills	6.7%	35.1%	23.6%	34.6%
Health literacy	3.6%	8.5%	26.7%	61.2%

Knowledge of disease was sufficient in 59% and the sense of disease severity sufficient in 69% of the parents.

#### Adherence and level of effort

Correlations were found between Dutch language skills and adherence (Spearman's rho 0.24, p < 0.01) and between health literacy and adherence (Spearman's rho 0.49, p < 0.01) in parents. Adherence was also correlated with knowledge of disease (Spearman's rho 0.47, p < 0.01) and sense of disease severity (Spearman's rho 0.53, p < 0.01).

Logistic regression showed that health literacy, knowledge of disease and sense of disease severity in the parents and not Dutch language skills, proved to be the best predictors for adherence (Table 3). The level of effort was adequately low (normal) in 14%, adequately high in 32%, inadequately high in 48% and inadequately low in 6% of the parents. No differences in levels of effort were found; neither between the three ethnic groups nor between the three diagnostic categories.

**Table 3:** Predictors of adherence to treatment

	OR	95% CI	*p*
Dutch language skills	0.99	(0.60-1.65)	0.97
Health literacy	2.00	(1.03-3.90)	0.04
Knowledge of disease	2.42	(1.30-4.50)	0.00
Sense of disease severity	2.40	(1.50-3.83)	0.00

OR: Odds Ratio; CI: Confidence Intervals

### Use of hospital facilities

**Figure 1:**
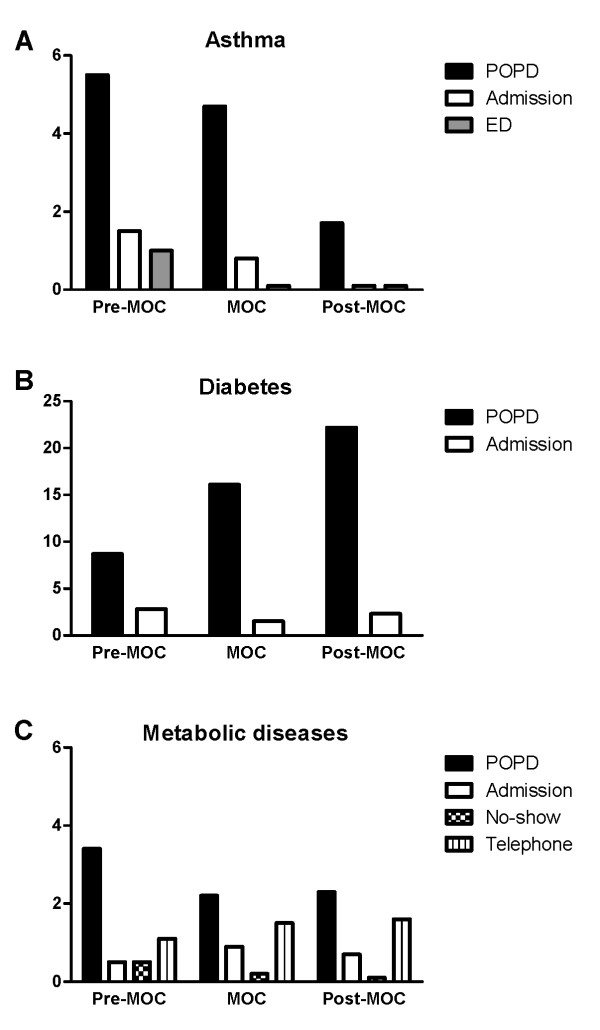
**Use of hospital facilities in patients with asthma, diabetes type 1 and metabolic disease before (2005), during (2006) and after (2007) attending the MOC**. MOC = Mosaic Outpatient Clinic; POPD = Paediatric Outpatient Department; ED = Emergency Department.

From the 119 patients in the three main diagnostic categories complete data on hospital use were available from only 85 patients (71%) in the year before (2005), during (2006) and after (2007) the start of the MOC; comprising 41 (70%) children with asthma, 19 (67%) with diabetes and 25 (75%) with a metabolic disease, due to intercurrent changes in the electronic registration systems of the participating hospitals. The use of hospital facilities is described per diagnostic category, i.e. asthma, diabetes type I and metabolic disease in Figure 1.

#### Patients with asthma (n = 41)

The mean number of POPD visits decreased from 5.5 in 2005 to 1.7 in 2007 (mean difference 3.80, SE 0.62; p = 0.00). The mean number of admissions was 1.51 in 2005 compared with 0.09 in 2007 (mean difference 1.43, SE 0.57; p = 0.05) and the mean number of ER visits was 1.0 in 2005 compared with 0.1 in 2007 (mean difference 0.86, SE 0.37; p = 0.08).

#### Patients with diabetes type 1 (n = 19)

The mean number of visits to the POPD increased from 8.7 in 2005 to 22.2 in 2007 (mean difference 13.47, SE 2.87; p = 0.00). The mean number of admissions did not change over time and was respectively 2.8 in 2005 and 2.3 in 2007 (F (2, 17) 0.53; p = 0.60). No ED visits were made from 2005 to 2007 as diabetic patients were always seen in the Paediatric Department, also after hours.

#### Patients with a metabolic disease (n = 25)

The mean number of POPD visits was 3.4 in 2005, 2.2 in 2006 and 2.3 in 2007 (F (2, 23) 3.18; p = 0.06). The number of admissions and telephone consultations did not differ between the years before and after Mosaic intervention was given. However, the amount of no-shows decreased between 2005 and 2007 from respectively 0.5 to 0.1 (mean difference 0.44, SE 0.16; p = 0.04).

## Discussion

There is a paucity of evidence-based data on appropriate health care interventions in vulnerable ethnic minority children and the challenge on how to deliver optimal health care to these children and their families has become an important issue in many industrialized countries [8,14-16].

This study describes the MOC, an integrated and patient-centred 2^nd ^-line outpatient clinic in ethnic-cultural diversity, to analyse bottlenecks in the health care for chronic sick minority children and their parents in Amsterdam, the Netherlands. It shows that it was possible, within one year, to integrate the MOC into the POPD's of two Teaching and one University hospital. The structure of the MOC with an initial inventory of the bottlenecks in the health care process was not only accepted but also much appreciated in the great majority of patients and parents. Moreover, the concept of the MOC proved to be reproducible since a similar initiative has recently been successfully introduced in a District Teaching Hospital in The Hague.

Our approach is not categorical but supportive for the paediatricians in the POPD. By referring the patient back to their own paediatrician with a written report we hope to increase their knowledge in managing diversity and insight in the specific health care problems of chronic sick ethnic minority children.

Functional health literacy represents the cognitive and social skills which determine the motivation and ability of individuals to gain access to, understand and use information in ways which promote and maintain good health [17]. Although the term health literacy, widely used in the USA, suggests that health professionals have the 'one and only' knowledge and that patients who have other concepts about health are 'illiterate', our patient-centered and participatory approach resulted in increased satisfaction of the parents in the health care given. Health literacy proved to be high despite poor Dutch language skills in a great number of the parents and although Turkish parents showed poorer language skills than Moroccan parents, functional health literacy was equal in both groups. Especially in chronic sick children adequate care and optimal adherence to treatment are warranted. Contrary to what one might expect, poor adherence was not associated with poor skills in Dutch language but with low functional health literacy, poor knowledge of disease and a limited sense of disease severity. It seems therefore important for a better adherence to promote health literacy and knowledge of disease in parents.

In a recent Dutch study on predictors of asthma control in ethnic minority children, it was found that command of the Dutch language is an important prerequisite for asthma control. However, other factors must also be involved as the Surinamese patients in that study, who spoke fluently Dutch, proved to be most at risk for uncontrolled asthma [9]. As no distinction was made between Dutch language skills and functional health literacy in that study comparison with our results is difficult.

Levels of effort in relation to the severity of the disease were adequate in nearly half of the parents. A great number of parents however, showed an inadequate high level of effort, indicating that they were over-concerned about the disease of their child. Only a small group of parents seemed to be so incompetent to understand and manage the disease in their child that they showed an inadequate low effort. These findings suggest that the majority of parents are not incompetent but very well able to handle the chronic disease in their children.

Only in patients with asthma the number of admissions and unscheduled visits to the POPD was reduced after the MOC was launched. One of the possible explanations might be that the involvement of culture competent healthcare workers was responsible for this decrease [18]. However, also in time differences can appear as the same group was studied during three different years. In patients with diabetes and metabolic diseases the results are not clear although the much wanted number of scheduled visits of patients with diabetes to the POPD increased substantially and the number of no-shows decreased in patients with a metabolic disorder after introduction of the MOC. Metabolic disorders are very complex, which makes it important for these patients, like in patients with diabetes type 1, to show up regularly at their scheduled appointments. A reduction in the number of no-shows is therefore promising.

The present study however, does have substantial limitations. Despite the high interrater reliability of the student health care workers in the assessment of the profiles of the parents, a certain degree of subjectivity cannot be ruled out which limits the generalisation of the results. In addition, the lack of a socio-economically matched control group of patients with a Dutch background and a control group of minority patients with the same diseases at the regular POPD hampers the interpretation of the cultural specificity of our findings. Finally, our results concerning the use of hospital facilities are based on only a small number of patients.

## Conclusion

Integration of a specific patient-oriented clinic in ethnic-cultural diversity in the general Paediatric Outpatient Department is feasible and appreciated by the parents, provides more insight in the problems chronic sick ethnic-minority children and their parents face and shows promising directions for optimizing adherence in these children.

## Competing interests

The authors declare that they have no competing interests

## Authors' contributions

All authors contributed to the study. ND and BW designed the study and drafted the manuscript. DM performed the data analyses. MC and DB made substantial contributions to the study conception and data acquisition. All authors have read and approved the final manuscript.

## Pre-publication history

The pre-publication history for this paper can be accessed here:

http://www.biomedcentral.com/1472-6963/12/12/prepub
